# Polymorphic Variants of *TNFR2* Gene in Schizophrenia and Its Interaction with -308G/A *TNF-α* Gene Polymorphism

**DOI:** 10.1155/2018/8741249

**Published:** 2018-09-04

**Authors:** Renata Suchanek-Raif, Paweł Raif, Małgorzata Kowalczyk, Monika Paul-Samojedny, Krzysztof Kucia, Wojciech Merk, Jan Kowalski

**Affiliations:** ^1^School of Pharmacy with the Division of Laboratory Medicine in Sosnowiec, Medical University of Silesia, Katowice, Poland; ^2^Department of Medical Genetics, Jedności 8 Street, 41-200 Sosnowiec, Poland; ^3^Department of Biosensors and Biomedical Signals Processing, Silesian University of Technology, Faculty of Biomedical Engineering, Roosevelta 40 Street, Zabrze, Poland; ^4^School of Medicine in Katowice, Medical University of Silesia, Katowice, Poland; ^5^Department of Psychiatry and Psychotherapy, Ziołowa 45 Street, 40-635 Katowice, Poland

## Abstract

**Aim:**

Many data showed a role of inflammation and dysfunction of immune system as important factors in the risk of schizophrenia. The TNFR2 receptor is a molecule that adapts to both areas. Tumor necrosis factor receptor 2 (TNFR2) is a receptor for the TNF-*α* cytokine which is a strong candidate gene for schizophrenia. The serum level of TNFR2 was significantly increased in schizophrenia and associated with more severe symptoms of schizophrenia.

**Methods:**

We examined the association of the three single nucleotide polymorphisms (rs3397, rs1061622, and rs1061624) in *TNFR2* gene with a predisposition to and psychopathology of paranoid schizophrenia in Caucasian population. The psychopathology was measured by a five-factor model of the PANSS scale. We also assessed a haplotype analysis with the -308G/A of *TNF-α* gene.

**Results:**

Our case-control study (401 patients and 657 controls) revealed that the genetic variants of rs3397, rs1061622, and rs1061624 in the *TNFR2* gene are associated with a higher risk of developing schizophrenia and more severe course in men. However, the genotypes with polymorphic allele for rs3397 SNP are protective for women. The rs1061624 SNP might modulate the appearance of the disease in relatives of people with schizophrenia. The CTGG haplotype build with tested SNPs of *TNFR2* and SNP -308G/A of *TNF-α* has an association with a risk of schizophrenia in Caucasian population depending on sex. Our finding is especially true for the paranoid subtypes of schizophrenia.

## 1. Introduction

The accurate pathogenesis of schizophrenia is still unknown. A deregulation of immune processes and inflammatory mechanisms in the central nervous system is suggested as important for the biology of schizophrenia. Evidence from the genetic, biomarker, and imaging studies strongly supports the hypothesis. Cytokine abnormalities in particular are associated with the pathophysiology of schizophrenia [1]. TNF-*α* is one of the strongest candidates among proinflammatory cytokines. A significantly elevated level of TNF-*α* in the plasma of schizophrenia patients was found [2]. Its polymorphic variants, in particular -308G/A SNP, was associated with predisposition to schizophrenia [3].

TNF-*α* is an important modifier many of the processes in the CNS and plays a role in neurotransmission. It shows its activity through two receptors: TNFR1 and TNFR2 [4]. Both receptors are constitutively expressed by all neural cell types and have a region-specific reparative effect in brain, especially in the hippocampus and striatum [5, 6]. Those areas of the brain are damaged in schizophrenia. The brain levels both of TNF-*α* receptors are altered in the course of severe mental disorders [7]. In schizophrenia, a serum level TNFR2 receptor is increased [8, 9].

The TNFR2 receptor is an important factor to remyelination and promotes proliferation of oligodendrocyte progenitors [10, 11]. There were shown abnormalities in oligodendrocytes in schizophrenia [12]. Gene expression studies showed a reduction of oligodendrocyte-related and myelin-related genes in schizophrenia among which TNFR2 gene was significantly decreased [13]. It was shown that TNFR2 receptor is involved in the protection of dopaminergic cells against oxidative stress [14]. Oxidative stress is one of the factors involved in the manifestation of schizophrenia [15]. The association between the level of TNFR2 and severity of psychotic symptoms of schizophrenia was found as well [8, 16].

In the present work, we have focused on the three SNPs in *TNFR2* gene. The polymorphisms rs3397 (T/C) and rs1061624 (G/A) are located in the 3′-untranslated region (3′-UTR). An increase in the frequency of some alleles in the human TNFR2 gene has been correlated with decreased TNFR2 expression [17]. The rs1061624 SNP was also associated with a hippocampus volume in healthy adults [18]. The rs1061622 (T/G) SNP is located in exon 6 at 676 nucleotide of TNFR2 gene. A change T to G nucleotide produces amino acid substitution from methionine (Met) to arginine (Arg) at position 196 (Met196Arg). It was also observed that an activation of NF-*κ*B signaling is reduced by polymorphic allele G of TNFR2 receptor [19]. The NF-*κ*B signaling changes were found in a different brain region in schizophrenia [20].

The aim of the present work was to evaluate the potential association between three TNFR2 single nucleotide polymorphisms (SNPs) (rs3397, rs1061622, and rs1061624) and schizophrenia in a Caucasian population. We evaluated the distribution of genotypes, alleles, and haplotypes in a case-control study. We assessed whether these SNPs are associated with the psychopathology of schizophrenia, as well as any family history of schizophrenia or suicide attempts. We also evaluated the interaction with the polymorphic variants of -308G/ATNF-*α* in haplotype analysis.

## 2. Methods

### 2.1. Subjects

The study subjects (*n* = 1058) consisted of 401 patients and 657 healthy controls. All of the participants were of Caucasian Polish origin and lived in the Silesia region. The patients were recruited from in-patients being treated at the Clinic of Psychiatry in Katowice. The patients were diagnosed with schizophrenia according to Diagnostic and Statistical Manual of Mental Disorders, 4th Edition, Text Revision (DSM-IV-TR) criteria. The degree of the severity of the psychotic symptoms was evaluated by two independent psychiatrists using the positive and negative scale (PANSS). The results of PANSS were presented in a five-factor model encompassing factors of positive (POS), negative (NEG), disorganization (DIS), excitement (EXC), and emotional distress (EMO) symptoms. The age of onset was defined as the first appearance of positive psychotic symptoms. Exclusion criteria were the presence of depressive episodes and comorbid mental disorders including anxiety disorder, schizoaffective disorder, organic brain disease, or substance dependence. In addition, endocrine and autoimmune diseases constituted exclusion criteria. All of the patients were assessed to be capable of understanding the study and provided written consent before inclusion, and their anonymity was preserved. The 74 patients had attempted suicide. In all cases, suicide attempts were confirmed by family members or significant others as well as by medical records. The data on family burden were obtained from interview with family as well as by medical records.

The control group had been recruited from among volunteer blood donors. Exclusion criteria for controls were current psychiatric problems, any other neurological disorders, a family history of schizophrenia (verified during the interview), and chronic and acute physical illness such as an infection, autoimmune, or allergic diseases.

### 2.2. Genotyping

Genomic DNA was isolated from peripheral blood using QIAampDNA Blood Mini Kit (Qiagen, Valencia, CA). The SNPs were genotyped using the TaqMan 5′-exonuclease allelic discrimination assay; the assay ID (Thermo Fisher Scientific Inc.) of each SNP was C__8861228_20 for rs3397, C__8861232_20 for rs1061622, and C__8861229_10 for rs1061624.

### 2.3. Statistical Analysis

Descriptive variables are presented as mean ± standard deviation (SD). Qualitative data are expressed as percentage values. Differences in the allele, genotype, and haplotype frequencies were compared by the *χ*^2^ test. The two-way ANOVA and Tukey's HSD (honest significance test) post hoc test were used for comparisons of PANSS subscales and the age of onset. Homogeneity of variance was assessed by the Levene test. The extent of linkage disequilibrium (LD) expressed in terms of *D*′ and *r* coefficients, haplotype frequencies, and possible departure from the Hardy–Weinberg equilibrium (HWE) was estimated using the SNPStats software. Statistical calculations were performed by using the STATISTICA version 10 (http://www.statsoft.com) and SNPStats (http://bioinfo.inconcologia.net) and R-Studio. All results with *p* < 0.05 were accepted as statistically significant.

## 3. Results

### 3.1. Sample Characteristics

One thousand fifty-eight (*n* = 1058) subjects were enrolled in this study. Among the 401 patients, 159 (40%) were women and 242 (60%) were men; the mean age ± standard deviation (SD) was 41.3 ± 12.4 years. The mean ± SD of the PANSS scores for the positive, negative, disorganization, emotional distress, and excitement subscales were 23.1 ± 5.4, 23.2 ± 5.8, 31.7 ± 6.9, 22.1 ± 4.9, 20.5 ± 5.6, respectively. The mean age of the onset of the disease was 25.6 ± 6.8 years. The control group consisted of 657 unrelated subjects and included 315 (48%) women and 342 (52%) men. The mean age ± SD was 40.8 ± 8.6 years.

### 3.2. Comparison of Genotype and Allele Distributions between Patients and Controls

Table 1 shows the genotype and allele distribution of the three SNPs of the *TNFR2* gene among the patients and the controls. The allele distribution did not significantly departure from Hardy–Weinberg Equilibrium (HWE) neither among patients with schizophrenia nor for controls (*p* > 0.05). There were no statistically significant differences in the distribution of both genotypes and alleles between the patients and the control group for any of the SNPs that were analysed.

The multiple logistic regression model was used to assess the association between sex and risk of schizophrenia. For rs3397 SNP, the combinational genotype T/C-C/C appeared to have an increased risk of schizophrenia for men but a decreased risk for women. In men group, a higher risk of schizophrenia was also connected with the combination T/G-G/G genotypes for rs1061622 and the G/G-G/A genotype for rs1061624 (Table 2).

### 3.3. Linkage Disequilibrium and Haplotype Analysis

All three SNPs were in a weak linkage disequilibrium (LD). LD for rs3397 and rs1061624 was *D*′ = 0.35, *r* = 0.32; *p* < 0.001, for rs1061622 and rs1061624 was *D*′ = 0.47, *r* = −0.23; *p* < 0.001. The interaction analysis with a covariate sex revealed that the CGG haplotype in men was associated with a higher risk of schizophrenia compared to women with the same haplotype [OR = 3.24; 95% CI: 1.16–9.07; *p* < 0.05].

We made the haplotype analysis between the *TNFR2* and *TNF-α* -308G/A polymorphisms. The haplotype was formed by rs3397 (T/C), rs1061622 (T/G), rs1061624 (G/A), and -308 (G/A), respectively. We found that the CTGG haplotypes were associated with a high risk for men compared to women with the same genotype [OR = 2.8; CI: 1.13–6.91; *p* < 0.05]. In contrast, the CTGG haplotypes were associated with a lower risk for women compared to women with the most frequent haplotype TTGG [OR = 0.41; CI: 0.18–0.95; *p* < 0.05].

### 3.4. Age of Onset and PANSS Subscales

To find the association of the tested SNPs on the psychopathology of schizophrenia, we performed the ANOVA test. We showed that the rs1061622 SNP was associated with higher mean scores for PANSS scale. The men with the T/G genotype had more intense both EXC symptoms (*F* = 4.9, df(1); *p* < 0.05) and DIS symptoms (*F* = 3.7, df(1); *p* < 0.05) compared to the women with the T/T genotype. The genotypes of both rs3397 and rs1061624 SNPs did not show any statistical differences for PANSS subscales. We also did not find any statistical differences between the age of the onset of schizophrenia and the genotypes of all three tested SNPs (*p* > 0.05, data not shown).

### 3.5. Family History of Schizophrenia

There were 98 patients with a family history of schizophrenia, mean age 41.3 ± 12.1 years. The mean age of onset was 25.5 ± 6.3 years. There were 61 (53%) men (mean age: 37.0 ± 11.5 years) and 37 (47%) women (mean age: 48.0 ± 9.7 years) in this group. We have found statistically significant difference between patients with and without family history of schizophrenia for rs1061624 polymorphism [G/G- 33 (34%) vs 105 (35%); G/A- 55 (56%) vs. 133 (44%); A/A- 10 (10%) vs. 65 (21%), *χ*^2^ = 7.39, *p* < 0.05, respectively]. The frequency A/A genotype was higher in the patients without family history of schizophrenia.

The multiple logistic regression overdominant models were showed that the G/A genotype is associated with the risk of schizophrenia [OR = 1.79; 95% CI: 1.03–2.58; *p* < 0.05] among patients that had relative with schizophrenia. Whereas, the A/A genotype was shown as protective for patients without relative with schizophrenia [OR = 0.42; 95% CI: (0.21–0.85), *p* < 0.01]. Haplotype analysis showed that the CTG haplotype was more frequent in patients with relative with schizophrenia than those ones without (12% vs. 5%, respectively) and was associated with a risk of schizophrenia [OR = 3.08, 95% CI: 1.26–8.31; *p* < 0.01].

### 3.6. Suicide Attempts

Among the 401 patients, 74 (19%) had attempted suicide, mean age ± SD 38 ± 10.9 years. The mean age ± SD of disease onset was 24.4 ± 5.7 years. There were 47 (64%) men (mean age ± SD 36.8 ± 10.4 years) and 27 (36%) women (mean age ± SD 41.4 ± 11.4 years) in this group. There were no statistically significant differences in the distribution of the genotypes, alleles, or haplotypes among the patients who had or had not attempted suicide for either the total sample or when stratified according to sex (data not shown, *p* > 0.05).

## 4. Discussion

We have conducted a case-control study of three SNPs in the *TNFR2* gene in a Caucasian population. Our results suggest that rs3397, rs1061622, and rs1061624 SNPs are associated with a risk of schizophrenia in men. A higher risk was found for the genotypes with polymorphic (rare) allele (MR+RR) both the rs3397 and rs1061622 SNPs. In turn, this combinational genotype of the rs3397 SNP was found as protective for the women. The rs1061624 SNP showed a high risk at the major homozygote with heterozygote (MM+MR). Our results are in accordance with a previous study that assessed the rs1061622 SNP in the susceptibility to schizophrenia. Similar to our findings, Thabet et al., 2011, found that the genotypes including polymorphic allele (T/G+G/G) were independent risk factors for schizophrenia and the association was especially true for paranoid subtype of this disorder. No significant differences were seen between disorganized or undifferentiated schizophrenia (Thabet et al., 2011). Our patient group included only paranoid subtype of schizophrenia as well. There have been no any data evaluating the frequency genotypes of polymorphisms rs3397 and rs1061624 in schizophrenia.

A previous study showed that a serum level TNFR2 receptor (sTNFR2) was related to global functioning in schizophrenia [8]. For this reason, we conducted the analysis of association between tested SNPs of *TNFR2* gene and course of schizophrenia. We used the five-factor PANSS which represents a complex factor model and is more stable than previous models (van der Gaag et al., 2006). Our analysis of psychopathology revealed that the T/G genotype of rs1061622 SNP was associated with more intense both disorganization (DIS) and excitement (EXC) symptoms in men. To the best of our knowledge, there have not been studies assessing polymorphic sites of *TNFR2* gene (rs3397, rs1061622, and rs1061624) and severity of paranoid schizophrenia. In our previous study, we found that polymorphic sites of gene receptor 1 of tumor necrosis factor (*TNFR1*) also were connected with excitement symptoms in paranoid schizophrenia (Suchanek-Raif et al., 2017). The excitement symptoms (included items such as excitement, hostility, impulsivity, and uncooperativeness) may affect the risk of aggressive behaviour and severity of psychopathological symptoms [21]. Sex is an important factor influence on symptoms and course of schizophrenia; however, the mechanism of the effect has not been known. It was revealed that the changes in both microstructure and function of the brain between men and women were associated with the varied presentation of aggression, impulsivity, and excitement in schizophrenia [22–24]. It was shown that genetic variants of TNFR1 and TNFR2 are associated with the striatal and hippocampus morphology in healthy people [18]. It is worth noting that there have been revealed participation of tested SNPs of TNFR2 gene in the modulation of symptoms of the central nervous system which coexist with other diseases. The genotypes with polymorphic allele (T/C, C/C) of rs3397 SNP were correlated with decreased cognitive function in cancer patients receiving high doses of morphine (Kurita et al., 2016). The additive effect polymorphic alleles of rs1061622 of *TNFR2* gene and other immune-response genes may modify coexisting symptoms like depressed mood, pain, and fatigue in cancer patients (Reyes-Gibby et al., 2013). Considering these data, the receptor TNFR2 along with TNFR1 could be one of the factors modulating the presentation of excitement symptoms of schizophrenia depending on sex.

We have not found any association-tested SNPs of *TNFR2* gene with the age of onset schizophrenia or suicide attempts. It was shown that the G/G genotype of rs3397 SNPs was associated with the adult onset paranoid schizophrenia, the first episode was above 22 years. No differences in genotype distributions were seen in patients with early onset of paranoid schizophrenia (first episode ≤ 21 years) (Thabet et al., 2011). Suicide risk is higher in schizophrenia, especially during first years after diagnosis (Carlborg et al., 2010). Data have shown the altered levels of many cytokines in the suicide attempters. Several papers revealed that TNF-*α* was increased in suicide victims [25, 26]. Our haplotype analysis showed the CGG haplotype formed by the rare alleles both rs3397 and rs1061622, and the dominate allele of rs1061624 was connected with a high risk of schizophrenia in men (OR = 3.2) compared to women. Additionally, we evaluated the haplotype build with the polymorphism of *TNF-α* gene at place -308G/A. The -308G/A SNP is the most intensively evaluated polymorphism of *TNF-α* gene. It was shown that the polymorphic variants of this SNP were associated with a changed level of *TNF-α* gene expression [27]. Association of studies of -308G/A *TNF-α* polymorphism with schizophrenia has produced discrepant results [3]. However, the meta-analysis revealed two important findings. First, genotypes of -308G/A SNP of *TNF-α* gene showed the sex differences in predisposition to schizophrenia. Secondly, the association became more specific for the paranoid subtype of schizophrenia [3]. Our previous study that has been under review revealed a similar interaction. In the current analysis, we showed that the haplotype build with polymorphism variants of *TNFR2* and *TNF-α* has an association with the risk of paranoid schizophrenia in Caucasian population depending on sex.

A risk of schizophrenia is increased among the relatives of affected persons, especially among the first-degree relatives. It is suggested that the family transmission of schizophrenia is based on genetic background rather than shared environment. Heritability of schizophrenia was estimated to be up to 70–80% [28]. We found that genotypes of rs1061624 TNFR2 gene may modify a development of schizophrenia among relatives of person with this disorder. The G/A genotype and CTG haplotype are associated with a risk of schizophrenia among patients that had relative with schizophrenia. Whereas, the A/A genotype was shown as protective for patients without relative with schizophrenia (OR = 0.42). To the best of our knowledge, this type of analysis was conducted in a Caucasian population for the first time. However, these results should be treated with caution due to the small number of the study group. It is worth noted that in healthy people, the G/G genotype of rs1061624 was associated with increased hippocampal volume relative to A/A and G/A genotypes. The volume of the hippocampus is reduced in the course of schizophrenia. It was found that the first-degree relatives of patients with schizophrenia had a volume reductions in the amygdala-hippocampal region and thalamus compared to control subjects [29].

## 5. Conclusion

Our research revealed that the genetic variants of rs3397, rs1061622, and rs1061624 in the *TNFR2* gene are associated with a higher risk of developing schizophrenia and more severe course in men. However, the genotypes with polymorphic allele for rs3397 SNP are protective for women. The rs1061624 SNP might modulate the appearance of the disease in relatives of people with schizophrenia. The CTGG haplotype build with tested SNPs of *TNFR2* and SNP-308G/A of TNF-*α* was associated with an increased risk of schizophrenia in men and a reduced risk in women. Our findings are especially true for paranoid subtypes of schizophrenia.

## 6. Limitations

Our study needs replication in larger groups especially for the analyses in subgroups such as suicide attempts and family history of schizophrenia.

## Figures and Tables

**Table 1:** Genotype and allele distributions for the *TNFR2* (rs3397, rs1061622, and rs1061624) polymorphisms in paranoid schizophrenia patients (*n* = 401) and control subjects (*n* = 657).

Polymorphisms	*N* (%)								
	Genotype					Allele			
*rs3397*	T/T	T/C	C/C	*χ* ^2^	*p*	T	C	*χ* ^2^	*p*
Patients	158 (39)	186 (47)	57 (14)	*0.62*	*0.73*	502 (63)	300 (37)	*0.36*	*0.54*
Control	243 (37)	318 (48)	96 (15)			804 (61)	510 (39)		
*rs1061622*	T/T	T/G	G/G	*χ* ^2^	*p*	T	G	*χ* ^2^	*p*
Patients	229 (57)	150 (38)	22 (5)	*0.40*	*0.81*	608 (76)	194 (24)	*0.14*	*0.70*
Control	387 (59)	233 (35)	37 (6)			1007 (77)	307 (23)		
*rs1061624*	G/G	G/A	A/A	*χ* ^2^	*p*	G	A	*χ* ^2^	*p*
Patients	138 (34)	188 (47)	75 (19)	*0.31*	*0.85*	464 (58)	338 (42)	*0.65*	*0.41*
Control	220 (33)	305 (46)	132 (20)			745 (57)	569 (43)		

**Table 2:** Interaction analysis between polymorphisms of TNFR2 gene and sex on the risk of schizophrenia.

Genotype	Model	Sex	Control (*n*)	Schizophrenia (*n*)	OR (95% CI)	*p* value
*rs3397*						
T/T	Dominant	Female	104	69	1.00	
T/C-C/C		Female	211	90	0.64 (0.43–0.95)	*p* < 0.05
T/C-C/C	Dominant	Female	211	90	1.00	
T/C-C/C		Male	203	153	1.77 (1.28–2.44)	*p* < 0.05
*rs1061622*						
T/T	Dominant	Female	191	99	1.00	
T/G-G/G		Male	124	60	1.48 (1.05–2.09)	*p* < 0.05
T/G-G/G	Dominant	Female	124	60	1.00	
T/G-G/G		Male	146	112	1.60 (1.07–2.35)	*p* < 0.05
*rs1061624*						
G/G-G/A	Recessive	Female	248	122	1.00	
G/G-G/A		Male	277	204	1.50 (1.13–1.99)	*p* < 0.05

## Data Availability

The data used to support the findings of this study are available from the corresponding author upon request.
